# Chloride Permeability Coefficient Prediction of Rubber Concrete Based on the Improved Machine Learning Technical: Modelling and Performance Evaluation

**DOI:** 10.3390/polym15020308

**Published:** 2023-01-07

**Authors:** Xiaoyu Huang, Shuai Wang, Tong Lu, Houmin Li, Keyang Wu, Weichao Deng

**Affiliations:** 1 School of Civil Engineering, Architecture and The Environment, Hubei University of Technology, Wuhan 430068, China; 2 Wuhan Construction Engineering Company Limited, Wuhan 430056, China

**Keywords:** machine learning, rubber concrete, prediction, algorithm, chloride permeability coefficient

## Abstract

The addition of rubber to concrete improves resistance to chloride ion attacks. Therefore, rapidly determining the chloride permeability coefficient (*D_CI_*) of rubber concrete (RC) can contribute to promotion in coastal areas. Most current methods for determining *D_CI_* of RC are traditional, which cannot account for multi-factorial effects and suffer from low prediction accuracy. Machine learning (ML) techniques have good non-linear learning capabilities and can consider the effects of multiple factors compared with traditional methods. However, ML models easily fall into the local optimum due to their parameters’ influence. Therefore, a mixed whale optimization algorithm (MWOA) was developed in this paper to optimize ML models. The main strategies are to introduce Tent mapping to expand the search range of the algorithm, to use an adaptive *t*-distribution dimension-by-dimensional variation strategy to perturb the optimal fitness individual to thereby improve the algorithm’s ability to jump out of the local optimum, and to introduce adaptive weights and adaptive probability threshold values to enhance the adaptive capacity of the algorithm. For this purpose, data were collected from the published literature. Three machine learning models, Extreme Learning Machine (ELM), Random Forest (RF), and Elman Neural Network (ELMAN), were built to predict the *D_CI_* of RC, and the three models were optimized using MWOA. The calculations show that the MWOA is effective with the optimized ELM, RF, and ELMAN models improving the prediction accuracy by 54.4%, 62.9%, and 36.4% compared with the initial model. The MWOA-ELM model was found to be the optimal model after a comparative analysis. The accuracy of the multiple linear regression model (MRL) and the traditional mathematical model is calculated to be 87.15% and 85.03%, which is lower than that of the MWOA-ELM model. This indicates that the ML model that is optimized using the improved whale optimization algorithm has better predictive ability than traditional models, providing a new option for predicting the *D_CI_* of RC.

## 1. Introduction

Concrete is one of the most widely used building materials today [1,2]. With the economic boom, the disposal of used tire rubber is becoming a significant issue for urban development [3]. Developing concrete from tire rubber is considered to be a viable technical solution contributing to resource and environmental protection [4,5,6,7,8,9,10]. With the rapid development of marine technology, concrete structures have been widely used in coastal projects which has led to widespread interest in the structural durability of concrete [11,12,13]. The structural durability of concrete plays a vital role in sustainable development. Chloride ion attack is one of the main factors affecting the durability of concrete structures [14,15,16,17]. Chloride ion attack can cause structural failure of concrete, which can lead to many problems. Rubber is a hydrophobic material and has a high resistance to permeation. Chloride ions are transported in concrete using water as a medium, so adding rubber can improve the concrete’s resistance to chloride ions [18,19,20,21,22]. The chloride permeation coefficient (*D_CI_*) is one of the three main indicators of concrete durability design, and its permeation process is also an effective way to understand chloride ion attack on concrete [23,24]. It is currently difficult to measure each project’s *D_CI_* and permeation rate of concrete [25]. To this end, several empirical models and equations have been developed to determine the *D_CI_* of concrete [26,27,28]. However, chloride permeation is a complex and time-consuming process, and these traditional methods cannot consider the influence of multiple factors and have low predictive accuracy [29,30]. Therefore, it is particularly important to find a quick and accurate method for determining the *D_CI_* of rubber concrete (RC).

Machine learning (ML) techniques have good non-linear learning capabilities, which can learn from given data and make accurate predictions through complex systems [31]. ML technology has also been applied in the study of RC. For example, Nyarko et al. [10] used 457 sets of RC data to build a 9-3-2 deep neural network (DNN) model to predict the strength of RC and demonstrated that the DNN accuracy was high at 0.9779. Gupta et al. [32] successfully investigated the mechanical properties of rubber concrete at high temperatures using a multi-input and multi-output artificial neural network (ANN) model. Ly et al. [33] used DNN to predict the strength of RC successfully and found the best structure to be 12-16-14-3-1. However, existing research has mainly focused on the mechanical properties of RC. Research on the chloride penetration of RC is limited.

Compared to traditional feed-forward neural networks, Extreme Learning Machine (ELM) can set the input weights randomly and obtain the output weights by least squares. This network has a better generalization capability and faster iteration speed, as the entire iteration process is not required, while the probability of the local extremum and overfitting is lower [34,35]. Since the input weights are random, there is a problem with blind iteration and low accuracy [36]. The random forest (RF) model was proposed in 2018 [37]. The advantages of the RF model are controllable generalization error, fewer parameters to be adjusted, suitability for high-dimensional feature vector spaces, and protection against overfitting to a certain degree. However, its parameters still have randomness and limitations [2,38]. Elman neural network (ELMAN) is similar to artificial neural networks in structure. The difference is that the ELMAN can store information. The output of the previous neuron is stored to guide the prediction of the next neuron. Therefore, the ELMAN has better dynamic prediction capability. However, due to the influence of weights and bias, it is prone to gradient explosion, resulting in lower prediction accuracy [39,40]. The three models have shown extraordinary capabilities in solving prediction problems and have been used in various studies [2,39,40,41,42], but the research applied to RC is still limited. Therefore, these three models were chosen for this study. 

Determining the ML model parameters is crucial, as they directly affect the model’s predictive performance [43]. The parameters can be optimized by intelligent algorithms [44]. Intelligent algorithms can improve the model’s predictive performance by searching for optimal parameters [45]. The main idea of the whale optimization algorithm (WOA) is to simulate the humpback whale foraging process, proposed in 2016. The WOA has fewer parameters and is also quite competitive compared to other optimization algorithms [46], so it is widely used in various fields [47,48,49]. However, the standard WOA suffers from slow convergence and local optimal solutions, so improvements are needed. Tent chaotic mappings have a more uniform distribution and are often used to optimize the initial populations of WOA [50,51]. Therefore, in this study, tent chaotic mapping was chosen to optimize the population of WOA. Since the linear weight adjustment strategy of WOA is not adapted to the constantly changing population, an adaptive weight adjustment strategy was introduced. The Probability threshold value was introduced to enhance the algorithm‘s global search ability [52]. At the end of the iteration, to avoid the WOA falling into the local optimum, an adaptive *t*-distribution dimension-by-dimensional variation strategy was used to perturb the optimal individual to increase the WOA’S ability to jump out of the local optimum [53]. There is limited research on applying improved algorithms to optimize ML models to predict *D_CI_* of RC, so this approach is used in this study.

In summary, this study chose the ELM, RF, and ELMAN models to predict the *D_CI_* of RC, while the WOA was improved to form a new mixed whale optimization algorithm (MWOA). The MWOA algorithm can improve the accuracy of machine learning models. The optimized model also has advantages over the traditional model. Thus, data were first collected from the published literature and created a database for analysis. Secondly, using the three models to predict the *D_CI_* of RC, the three models were optimized using MWOA. Thirdly, we evaluated the model performance and found the optimal model. Fourth, we conducted sensitivity analysis for models. Fifth, the representative prediction result of the optimal model was compared with the actual value. Finally, using the optimal model compared with the traditional model.

## 2. Database Description and Analysis of Variables

**Figure 1:**
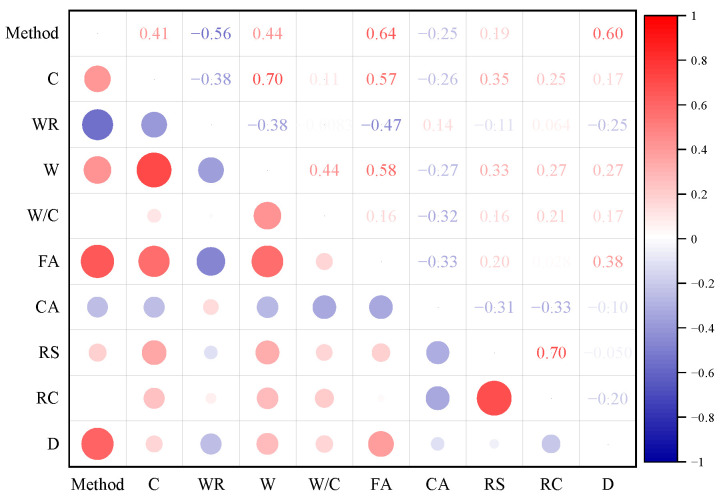
Heat map of correlation coefficients for input and output variables.

**Figure 2:**
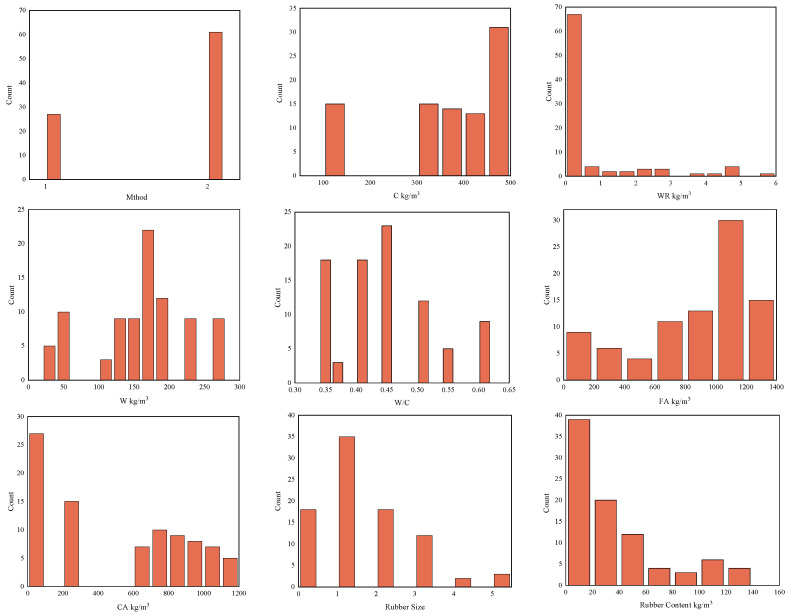
Histogram of frequency distribution for input and output variables.

Since the experimental data were collected from the published literature, processing is required to allow the model to learn better. This study collected 88 sets of RC mixed ratio data [22,54,55,56,57,58,59]. Three ML algorithms using nine input variables were used: (1) measurement method, (2) cement content, (3) water reducing agent content, (4) water content, (5) water to ash ratio, (6) fine aggregate content, (7) coarse aggregate content, (8) rubber size, (9) rubber content. The *D_CI_* is the only output variable. Due to the different methods used to measure the *D_CI_* of RC, this study distinguishes between the measurement methods. Two measurement methods are included in the collected literature, the rapid chloride permeability test (RCPT) method and the rapid chloride migration Test (RCM) method. Therefore, the RCPT method is defined as 1, and the RCM method is defined as 2. Considering the different types and sizes of rubber, this study distinguishes between rubber by size under different measurement methods. The RCPT literature method includes two rubber sizes, 0–1 mm and 1–3 mm. Therefore, the two rubbers were recorded as 1 and 2 by size. The RCM method literature includes five types of rubber with dimensions of 0.063–0.6 mm, 0.25 mm, 0.6–0.7 mm, 1–2 mm, and 4–10 mm. Therefore, the five types of rubber are noted as 1, 2, 3, 4, and 5 by size. The RC samples selected for this study met the 28-day curing period. Cement substitution materials were not used as an input variable in this study, as they are rarely added in the published literature. Figure 1 represents the hotspot plot of the correlation coefficient between the variables. The correlation coefficients between the variables are all less than 0.8, as seen from the graph. Some studies suggest that the correlation between variables should be less than 0.8 to reduce the effect of multiple collinearities [60,61]. Figure 2 represents the input and output parameters’ frequency distribution histogram. The statistical analysis of each variable is shown in Table 1. Stdd denotes overall sample bias, and Stde indicates sample bias.

## 3. Method

### 3.1. Whale Optimization Algorithm

Humpback whales are herd animals because they can only hunt small fish and prawns. They have developed a unique way of hunting known as bubble net hunting, where the term WOA comes from [46]. The algorithm is divided into three main parts: encirclement predation, prey predation, and prey search. The specific process of the WOA is as follows:

#### 3.1.1. Encirclement Predation

In this process, humpback whales randomly search for prey based on the positions of each other in the population. Since the location of the best target has yet to be discovered in the search space, the WOA assumes that the location of the best target is within the search range. Once the position of the best target is determined, other populations will approach the best target and update their positions. The mathematical expression for this process is as follows [62]:

(1) $$X\left(v+1\right)=X^{*}\left(v\right)-A\cdot D$$

(2) $$D=\left|CX^{*}\left(v\right)-X\left(v\right)\right|$$

where *v* indicates the number of iterations; *A* and *C* are vector coefficients; $X^{*}\left(v\right)$ is the location of the best target; *X(v)* is the current location; *D* is the process quantity; the expressions for the calculation of *A*, *C* are as follows [62]:

(3) $$A=2a\cdot r_{1}-a$$

(4) $$C=2r_{2}\;$$

(5) $$a=2-\frac{2v}{V_{max}}\;$$

where *v* indicates the number of iterations; $V_{max}$ indicates the maximum number of iterations; $r_{1}$ and $r_{2}$ are both random numbers belonging to the range [0, 1]; a decrease gradually from 2 to 0.

#### 3.1.2. Prey Predation

The bubble net foraging method is a unique hunting method for humpback whales. The WOA is a simulation of the spiral bubble net foraging strategy for optimization. A total of two methods were designed to simulate this behavior:

(1) Shrinkage envelope mechanism: This is achieved by reducing the value of a in Equation (3). Other targets will move closer to the best target when the best target is identified. The current position (*X*, *Y*) is gradually contracted to the optimal target position (X∗,Y∗).

(2) Spiral update mechanism: The distance between any whale (X,Y) and the optimal target position (X∗,Y∗) is first calculated, then spiral update equations are created to simulate the whale’s hunting motion. The main expressions are as follows [62]:

(6) $$D^{\prime }=\left|X^{*}\left(v\right)-X\left(v\right)\right|$$

(7) $$X\left(v+1\right)=D^{\prime }\cdot e^{bl}\cdot cos\left(2\pi l\right)+X^{*}\left(v\right)\;$$

where *b* is a constant and indicates the parameter for the shape of the spiral; l denotes a random number between [–1, 1]; $D^{\prime }$ suggests the distance between the best target and any whale.

These two mechanisms co-occur, with a probability of 50% each. The expression of the equation is as follows [62]:

(8) $$X\left(v+1\right)=\{\begin{array}{c} X^{*}\left(v\right)-A\cdot D\;\;\;\;\;\;\;\;\;\;\;\;\;\;\;\;\;\;\;P<0.5\\ X^{*}\left(v\right)+D^{\prime }\cdot cos\left(2\pi l\right)\cdot e^{bl}\;\;\;\;\;\;\;P\geq 0.5 \end{array}$$

#### 3.1.3. Prey Search

Whale populations randomly search for prey, and the mathematical expression for this process is as follows [62]:

(9) $$D_{rand}=\left|CX_{rand}\left(v\right)-X\left(v\right)\;\right|\;$$

(10) $$X\left(v+1\right)=X_{rand}\left(v\right)-A\cdot D_{rand}\;$$

where $X_{rand}\left(v\right)$ indicates a randomly selected location in the current population of whales.

The WOA, like other intelligent algorithms, suffers from the problem of falling into local extremum. Therefore, improvements to the WOA are needed.

### 3.2. Improved Whale Optimization Algorithm

#### 3.2.1. Tent Chaotic Mapping Initializes Populations

Chaos is a complex, non-linear state that exhibits irregularity and randomness [63]. Therefore, chaotic mapping can be used to improve the algorithm’s performance. The two commonly used chaotic mapping sequence models are Logistic and Tent. Compared to logistic mappings, Tent mappings have a more uniform distribution, allowing the algorithm to have a wider search range [50]. Therefore, Tent chaotic mapping is used to initialize the population in this study. The expressions are as follows [51]:

(11) $$x_{n+1}=\{\begin{array}{c} 2x_{n},\;\;\;\;\;\;\;\;0\leq x_{n}\leq 0.5\\ 2\left(1-x_{n}\right),\;\;0.5\leq x_{n}\leq 1 \end{array}$$

The expression after the Bernoulli displacement transformation is as follows [51]:

(12) $$x_{n}=2\left(x_{n}\right)mod1$$

#### 3.2.2. Adaptive Adjustment of Weight

The inertia weight is a crucial parameter in the WOA. Appropriate weight values can improve the algorithm’s performance, since the original WOA did not consider that the prey would guide the whale for position updates during the iterative process. Therefore, an adaptive weight formula is established in this paper. The specific expression is as follows [52]:

(13) $$w=d_{1}*\left(P_{worst}-P_{best}\right)+d_{2}*\left(x_{i}^{upper}-x_{i}^{lower}\right)/t$$

where *t* indicates the current number of iterations; $x_{i}^{upper}$ and $x_{i}^{lower}$ denote the upper and lower bounds of $x_{i}$ respectively; $d_{1}$ and $d_{2}$ represent constants; $P_{worst}$ and $P_{best}$ denote the worst and best positions of the current population respectively. Thus Equations (1) and (7) can be improved as:

(14) $$X\left(v+1\right)=w*X^{*}\left(v\right)-A\cdot D$$

(15) $$X\left(v+1\right)=D^{\prime }\cdot e^{bl}\cdot cos\left(2\pi l\right)+w*X^{*}\left(v\right)$$

With the introduction of an adaptive adjustment weights strategy, the algorithm can adaptively change the weights’ size according to the whale population’s current distribution. At the beginning of the algorithm iteration, if the whale population falls into the local optimum and the difference between the optimum and the worst solution is not significant, the value of $d_{2}*\left(x_{i}^{upper}-x_{i}^{lower}\right)$/*t* is not affected by the population distribution. At this point, obtaining a large value of weights is still possible and avoids the algorithm falling into a smaller search range at the beginning of the iteration. As the whale population iterations increase, the value of $d_{2}*\left(x_{i}^{upper}-x_{i}^{lower}\right)$/*t* decreases, and the effect on the weights decreases. If the algorithm does not obtain an optimal solution, $d_{1}*\left(P_{iworst}-P_{ibest}\right)$ can play a dominant role in the weight and can make the algorithm find the optimal solution in larger steps. The adjustment of these two components makes the inertia weights highly adaptive and strengthens the algorithm’s optimization search capability.

#### 3.2.3. Adaptive Adjustment of the Search Strategy

To prevent the algorithm from falling into the local optimum, a Probability threshold value *Q* is introduced to update the expression of the random search. The expression for *Q* is as follows [52]:

(16) $$Q=\frac{\left|\bar{f}-f_{min}\right|}{\left|f_{max}-f_{min}\right|}$$

where $\bar{f}$ indicates the average fitness of the current population; $f_{min}$ indicates the current best fit value; $f_{max}$ indicates the current worst fit value; for each whale, a q∈[0,1] is compared with *Q* value. If *q < Q*, the randomly selected individual whale updates its position according to Equation (17), and the other individual whales remain unchanged [52]. Otherwise, other individuals update their position according to Equation (10). This allows the algorithm to generate a set of random solutions globally with a greater probability in the early iterations, reducing the likelihood of population diversity decline and enhancing the global search capability of the algorithm.

(17) $$X_{rand}\left(v\right)=X_{min}+r*\left(X_{max}-X_{min}\right)$$

where r is a random number between [0, 1]; $X_{min}$ and $X_{rand}$ are the maximum and minimum values of $X_{rand}$ respectively.

#### 3.2.4. Adaptive *t*-Distribution Dimension-by-Dimensional Variation Strategy

**Figure 3:**
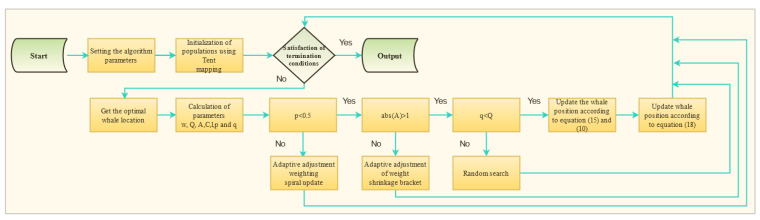
The flow chart of MWOA.

Population diversity declines in later iterations of the WOA. This leads to the algorithm being prone to fall into the local optimum. Therefore, this study introduces an adaptive *t*-distribution dimension-by-dimensional variation strategy to perturb the individuals with optimal fitness and improve the ability of the algorithm to jump out of the local optimum. Depending on the size of the degree of freedom *n*, the *t*-distribution curves show different patterns. When t→(n→∞)→N(0, 1), t(n=1)=C(0, 1), where *N* (0, 1) is a Gaussian distribution and *C* (0, 1) is a Cauchy distribution. This shows that the two boundary special cases of the *t*-distribution are the Gaussian and the Cauchy distributions [64]. The dimension-by-dimension variation is calculated as follows [53]:

(18) $$X_{new}^{d}=X_{Pest}^{d}+X_{Pest}^{d}\times t\left(iter\right)$$

where iter indicates the current number of iterations; t(iter) denotes *t*-distribution with the degree of freedom parameter *t.* The flow chart for the MWOA is shown in Figure 3.

### 3.3. Machine Learning Models

#### 3.3.1. Extreme Learning Machine

**Figure 4:**
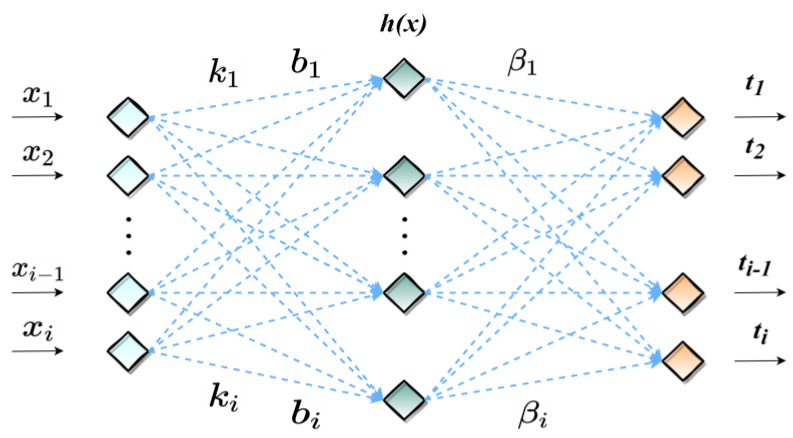
Schematic diagram of the ELM structure.

The Extreme Learning Machine is a new neural network learning algorithm proposed by Professor Guangbin Huang in 2004 [65]. The Extreme learning machine also evolved from the feedforward neural network, which can randomize the input weights, bias, and the number of hidden layer neurons and then obtain the output weights by least squares without the need for the entire iteration of the network [34,35]. ELM is widely used in various fields such as pattern recognition, image processing, signal processing, combinatorial optimization, and prediction [66,67,68,69]. The structure of the ELM is shown in Figure 4. The primary calculation process for ELM is as follows:

For *N* arbitrary samples $\left(x_{i},t_{i}\right)$, where $x_{i}={\left[x_{i1},x_{i2},x_{i3},\ldots ,x_{in}\right]}^{T}\in R^{n}$, $t_{i}={\left[t_{i1},t_{i2},t_{i3},\ldots ,t_{im}\right]}^{T}\in R^{m}$. Assume that the number of hidden layer neurons is $\tilde{N}$, and the activation function is h(x), the standard single hidden layer feedforward neural network (SLFN) expression is as follows [70]:

(19) $$\overset{\tilde{N}}{\underset{i=1}{\sum }}\beta_{i}h\left(k_{i}\cdot x_{j}+b_{i}\right)=y_{j},\;j=1,\ldots N$$

where $k_{i}={\left[k_{i1},k_{i2},k_{i3},\ldots ,k_{in}\right]}^{T}$ denote the vector of weights connecting the *i^th^* hidden neuron to the input neuron;$\;b_{i}$ denotes the bias of the *i^th^* neuron; $\beta_{i}={\left[\beta_{i1},\beta_{i2},\beta_{i3},\ldots ,\beta_{in}\right]}^{T}$ denotes the weights connecting the *i^th^* hidden layer neuron to the output neuron. $k_{i}\cdot x_{j}$ denote the inner product of $k_{i}$ and $x_{j}$; The activation function is usually Sigmoid, RBF or Sine, and in this study the activation function is Sigmoid.

A standard SLFN with $\tilde{N}$ hidden layer neurons and activation function h(x) can approach this *N* samples with zero error, where $\overset{\tilde{N}}{\underset{j=1}{\sum }}||y_{j}-t_{j}||=0$. Thus, the following expression exists [70]:

(20) $$\overset{\tilde{N}}{\underset{i=1}{\sum }}\beta_{i}h\left(k_{i}\cdot x_{j}+b_{i}\right)=t_{j},\;j=1,\ldots N$$

The above *N* equations can be written as [70]:

(21) Hβ=T

Where $H\left(k_{1},k_{2},k_{3},\ldots k_{\tilde{N}},b_{1},b_{2},b_{3},\ldots ,b_{\tilde{N}},x_{1},x_{2},x_{3},\ldots ,x_{n}\right)$

$$={\left[\begin{array}{ccc} h\left(k_{1}\cdot x_{1}+b_{1}\right) & \cdots & h\left(k_{\overset{ˇ}{N}}\cdot x_{1}+b_{\tilde{N}}\right)\\ \vdots & \ddots & \vdots \\ h\left(k_{1}\cdot x_{N}+b_{1}\right) & \cdots & h\left(k_{\tilde{N}}\cdot x_{N}+b_{\tilde{N}}\right) \end{array}\right]}_{N\times \tilde{N}},\;\beta ={\left[\begin{array}{c} \beta_{1}^{T}\\ \vdots \\ \beta_{3}^{T} \end{array}\right]}_{\tilde{N}\times m},\;T={\left[\begin{array}{c} t_{1}^{T}\\ \vdots \\ t_{N}^{T} \end{array}\right]}_{N\times m}$$.

*H* is called the hidden layer output matrix of the neural network [71,72]. The *i^th^* column of *H* is the output vector of the *i^th^* hidden neuron concerning the input $x_{1},x_{2},x_{3},\ldots ,x_{n}$.

When the input layer weights and the hidden layer bias are determined, the hidden layer output matrix *H* can be obtained by following the input samples. So, the final conversion is to find the least squares solution for Hβ=T [70]:

(22) $$||H\left(K_{1},\ldots ,K_{\tilde{N}},b_{1},\ldots ,b_{\tilde{N}}\right)\hat{\beta }-T||=\underset{\beta }{\min}H\left(K_{1},\ldots ,K_{\tilde{N}},b_{1},\ldots ,b_{\tilde{N}}\right)\beta -T$$

the least squares solution of Equation (14) is as follows [70]:

(23) $$\hat{\beta }=H^{†}T$$

where: $H^{†}$ is the Moore-Penrose generalized inverse matrix of the matrix *H* [73].

Random input weights and hidden layer bias can lead to problems, such as blind iterations and accuracy degradation [36]. Therefore, this study introduces the MWOA into the ELM model to optimize the input weights and hidden layer bias to improve the model’s accuracy.

#### 3.3.2. Random Forest Model

The RF model is one of the most commonly used regressions and classification models proposed by Leo Breiman in 2001 [74]. The main idea is to train decision trees by taking *n* samples from the original data set *N* to form a new training set, and *m* random forests are created by these *n* decision trees at random [75,76]. Meanwhile, the predicted value is decided by the voting of these *m* random forests [77]. A mathematical model can explain the RF regression model, the leading theory being that *X* is the independent variable (input data) and *Y* is the dependent variable (output data). Assuming that the distributions of (*X*, *Y*) are independent, the randomly generated training set is *Q,* and the predicted outcome is *G(X)*, the mean squared generalization error is [78]:

(24) $$E_{X,Y}{\left[Y-G\left(X\right)\right]}^{2}$$

Assuming that there are *h* decision trees, the average of the predicted values $\left\{G\left(Q,X_{h}\right)\right\}$ of the *h* decision trees is the prediction of the RF regression. If h→∞, then the following equation holds [78]:

(25) $$E_{X,Y}{\left[Y-\bar{G_{h}}\left(X,Q_{h}\right)\right]}^{2}\to E_{X,Y}{\left[Y-E_{Q}\left(X,Q_{h}\right)\right]}^{2}$$

where $E_{X,Y}{\left[Y-E_{Q}\left(X,Q_{h}\right)\right]}^{2}$ denotes the generalization error, noted as *M*. When *h* is infinite, the average generalization error of a single tree is noted as $M^{*}$. The expression for $M^{*}$ is as follows [78]:

(26) $$M^{*}=E_{Q}E_{X,Y}{\left[Y-G\left(X,Q\right)\right]}^{2}$$

where *Q* satisfies the following expression [78]:

(27) $$M\leq \bar{\rho }M^{*}$$

where $\bar{\rho }$ denotes the residual weighted correlation coefficient. The final RF regression function is as follows [78]:

(28) $$Y=E_{Q}G\left(X,Q\right)$$

**Figure 5:**
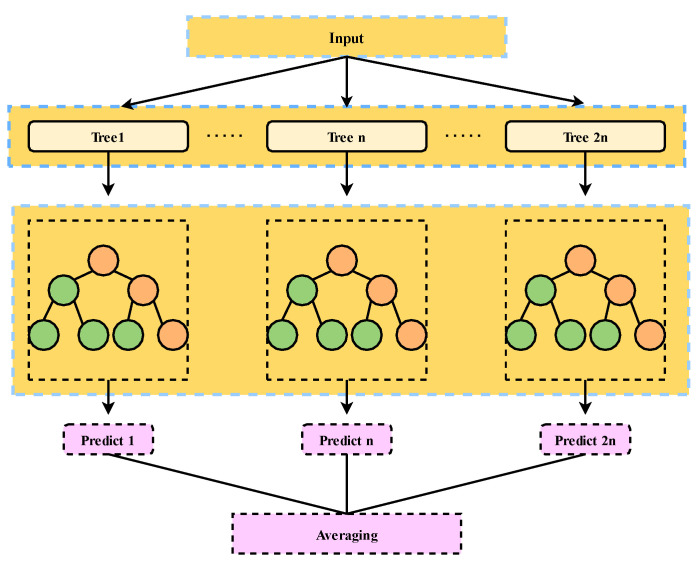
The structure of RF model.

Since the number of forests and leaves in the RF model significantly impacts the model’s performance, at the same time, they have randomness and limitations. Therefore, this study introduces MWOA to optimize these two parameters. The structure of the RF model is shown in Figure 5.

#### 3.3.3. ELMAN Neural Network

**Figure 6:**
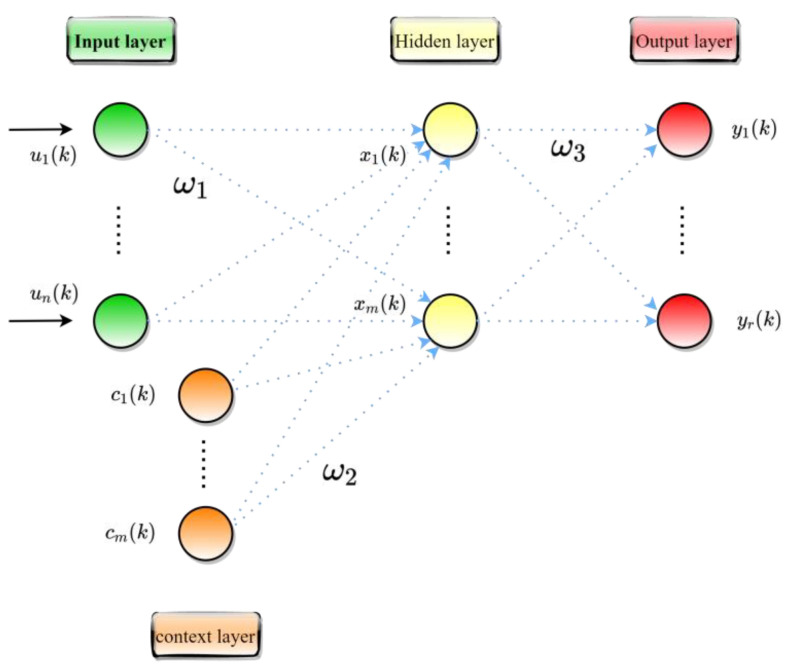
The structure of ELMAN.

Elman neural network was proposed by ELMAN in 1990 [79]. ELMAN is a multi-layer dynamic recurrent neural network that can approximate nonlinear functions well and is therefore used in many industries [39,80,81]. Like artificial neural networks, ELMAN has an input, hidden, and output layer. The difference is that ELMAN has a unique storage layer. This particular storage layer, which acts as a delay operator, can store the output values of the neurons in the previously hidden layer, giving the network a memory function and improving the net work’s ability to process dynamic information. The structure of ELMAN is shown in Figure 6. The expression for ELMAN at the moment *t* is as follows [82]:

(29) $$x_{j}\left(k\right)=f\left(\overset{n}{\underset{i=1}{\sum }}\omega 1_{i,j}u_{i}\left(k\right)+\overset{m}{\underset{i=1}{\sum }}\omega 2_{i,j}c_{i}\left(k\right)\right)$$

(30) $$c_{i}\left(k\right)=x_{i}\left(k-1\right)$$

(31) $$y_{j}\left(k\right)=g\left(\overset{r}{\underset{i=1}{\sum }}\omega 3_{i,j}x_{i}\left(k\right)\right)$$

where $\omega 1_{i,j}$ denotes the weight of node *i* in the connected input layer and node *j* in the hidden layer; $\omega 2_{i,j}$ denotes the weight of node *i* and node *j* in the connection storage layer; $\omega 3_{i,j}$ denotes the weight connecting node *i* in the hidden layer and node *j* in the output layer; $x_{j}\left(k\right)$, $c_{i}\left(k\right)$ and $y_{j}\left(k\right)$ denote the output vectors of the hidden layer, the storage layer and the output layer respectively. *f* and *g* denote the transfer functions of the hidden layer and the output layer, respectively. The transfer function for this study is tanh.

ELMAN calculates the number of hidden layer neurons in the same way as ANN. The main expressions are as follows [31]:

(32) $$h=\sqrt{m+n}+a$$

where *m* is the number of nodes in the input layer; *n* is the number of nodes in the output layer; a∈(1, 10).

ELMAN’s predictive performance is influenced by weights and biases. Therefore, the optimal weights and biases are found by optimizing the ELMAN neural network using MWOA.

**Figure 7:**
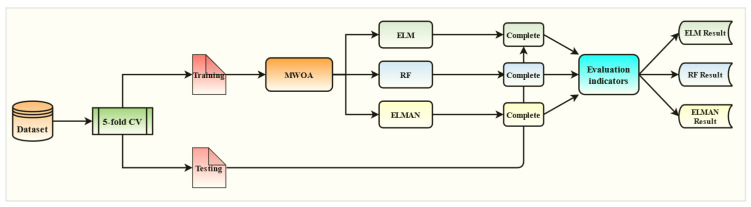
Flow chart of the research process.

The flow chart of the research process is shown in Figure 7.

## 4. Evaluation Indicators for the Three Models

This study uses root mean square error (RMSE), mean absolute error (MAE), mean absolute percentage error (MAPE), and coefficient of determination (*R*^2^) to assess the performance of the model. *R*^2^ is the metric used to evaluate the accuracy of the model’s predictions [83,84]. The closer the *R*^2^ value is to 1, the closer the MAE is to 0, and the more accurate the model will be. These four evaluation indicators are expressed as follows [85,86]:

(33) $$R^{2}=\frac{\sum_{k=1}^{N}\left(q_{0,k}-\bar{q_{0}}\right)\left(q_{t,k}-\bar{q_{t}}\right)}{\sqrt{\sum_{k=1}^{N}{\left(q_{0,k}-\bar{q_{0}}\right)}^{2}\sum_{k=1}^{N}{\left(q_{t,k}\sum \bar{q_{t}}\right)}^{2}}}\;$$

(34) $$MAE=\frac{1}{N}\left(\overset{N}{\underset{K=1}{\sum }}\left|\frac{q_{0,k}-q_{t,k}}{q_{0,k}}\right|\right)\;$$

(35) $$RMSE=\sqrt{\frac{1}{N}\overset{N}{\underset{k=1}{\sum }}{\left(q_{0,k}-q_{t,k}\right)}^{2}}\;$$

(36) $$MAPE=\frac{100\%}{N}\overset{N}{\underset{k=1}{\sum }}\left|\frac{q_{t}-q_{0}}{q_{0}}\right|$$

where *N* indicates the number of samples; *q*_0_ indicates the actual value; $\bar{q_{0}}$ indicates the actual average value; $q_{t}$ indicates the output value; $\bar{q_{t}}$ indicates the output average value, *k =* 1:*N*.

## 5. Results of the Three Models

The objective of the computational analysis was to predict the $D_{CI}$ of the RC using three ML models (MWOA-ELM, MWOA-RF, and MWOA-ELMAN). Therefore, the model optimized by WOA and the conventional model was also built for comparison. A cross-validation operation is also used in the calculation process, and the result is the average of a 5-fold cross-validation. This was done to make the results more realistic and to avoid chance. Therefore, the data set is divided into five groups by a 5-fold cross-validation operation. For each training session, one set was used as the testing set and the remaining four sets were used as the training set. This resulted in three 70 training sets, 18 test sets, two 71 training sets, and 17 test sets of data sets. Consistent data for each model during training and testing by programming. The models constructed and the computational results are described in detail in the following subsections.

### 5.1. MWOA-ELM Model Result

Like neural network models, ELM models need to determine the number of hidden layer neurons. In this study, the number of neurons in the hidden layer of the ELM model was calculated by the corresponding program and determined by the trial-and-error method to be 28. The parameter settings for the MWOA-ELM model in this study are shown in Table 2. The parameter settings for the WOA-ELM model are the same as the MWOA-ELM model. 

**Figure 8:**
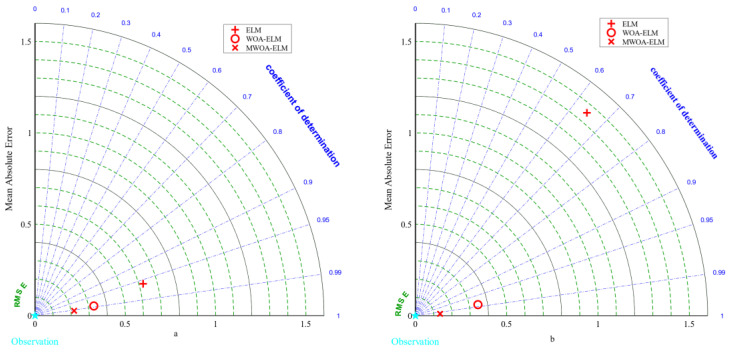
Taylor diagrams for the Training (**a**) and Testing (**b**) sets of the three ELM models.

The average results of the three ELM models under 5-fold cross-validation are presented in Table 3. It was clear that the MWOA-ELM model performs the best. Its test set $R^{2}$ improved from 0.6458 to 0.9971, while the other error metrics RMSE, MAE, and MAPE were all the lowest among the three ELM models. Figure 8a,b represent the Taylor diagrams for the training and testing sets of the three ELM models [87]. As can be seen from the graph, the MWOA was effective, as reflected by the fact that the MWOA-ELM model was closest to the optimal reference point for each indicator.

### 5.2. MWOA-RF Model Result

Introducing the MWOA is to find the optimal number of forests and leaves for the RF model. The parameter settings for the MWOA-RF model for this study are shown in Table 4. The parameter settings for the WOA-RF model are the same as the MWOA-RF model.

**Figure 9:**
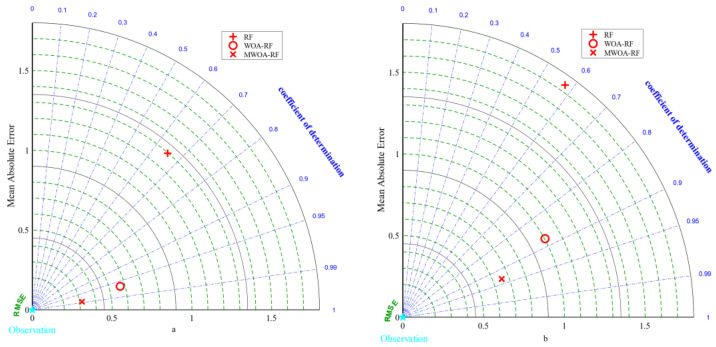
Taylor diagrams for the Training (**a**) and Testing (**b**) sets of the three RF models.

The results of the 5-fold cross-validation of the three random forest models are presented in Table 5. On the test set, the MWOA-RF model had the highest *R*^2^ of 0.9341 and the lowest values of 1.0164, 0.6533, and 0.0962 for RMSE, MAE, and MAPE, respectively. Figure 9a,b show the Taylor diagrams for the three RF models on the training and testing sets. It was clear that the MSSA-RF model was closest to the optimal reference point among the three evaluation indicators of the Taylor diagram. Therefore, MWOA is effectively increased the probability of the RF model finding the optimal number of forests and leaves.

### 5.3. MWOA-ELMAN Model Result

A three-layer feed-forward MWOA-ELMAN model was established, and the optimal number of neurons was obtained by Equation (32) and trial-and-error method as 13. The MWOA-ELMAN model parameter settings for this study are shown in Table 6. The parameter settings for the WOA-ELMAN model are the same as the MWOA-ELMAN model.

**Figure 10:**
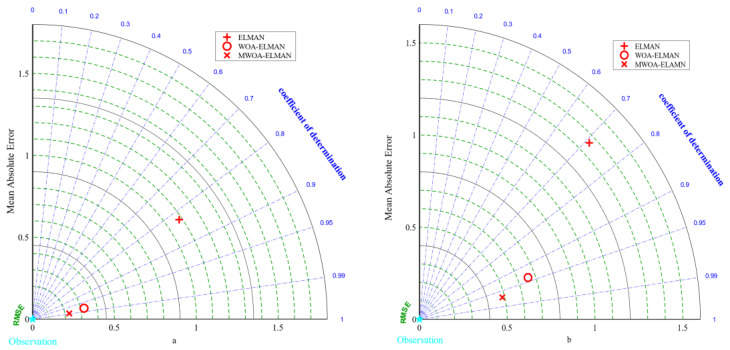
Taylor diagrams for the Training (**a**) and Testing (**b**) sets of the three ELMAN models.

The 5-fold cross-validation results for the three ELMAN models are presented in Table 7. Similar to the pattern of the first two models, the MWOA-ELMAN model has the best results. Figure 10 represents the Taylor diagrams for the training and testing sets. From Figure 10, the results of the MWOA-ELMAN model were closest to the optimal reference point, indicating that the algorithm is effective in optimizing the weights and improving the model’s prediction accuracy.

## 6. Discussion

### 6.1. Comparative Analysis of the Three Models

**Figure 11:**
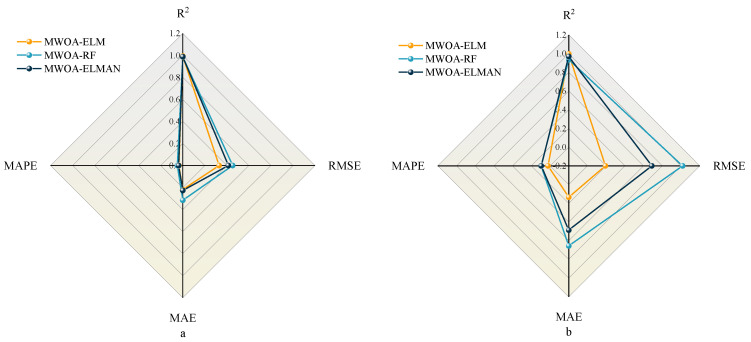
Radar plots of metrics for the Training (**a**) and Testing (**b**) sets for MWOA-ELM, MWOA-RF, and MWOA-ELMAN models.

**Figure 12:**
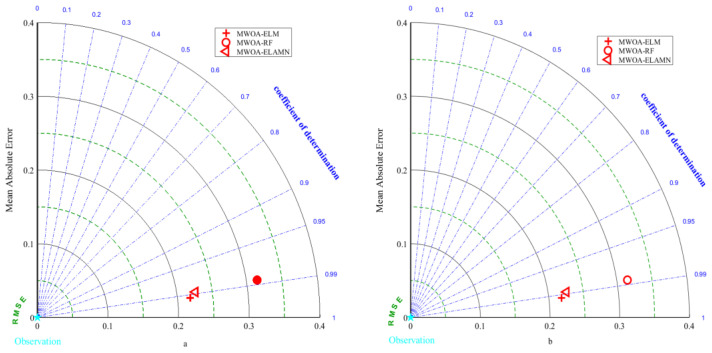
Taylor diagrams for the Training (**a**) and Testing (**b**) sets of the MWOA-ELM, MWOA-RF and MWOA-ELMAN models.

**Figure 13:**
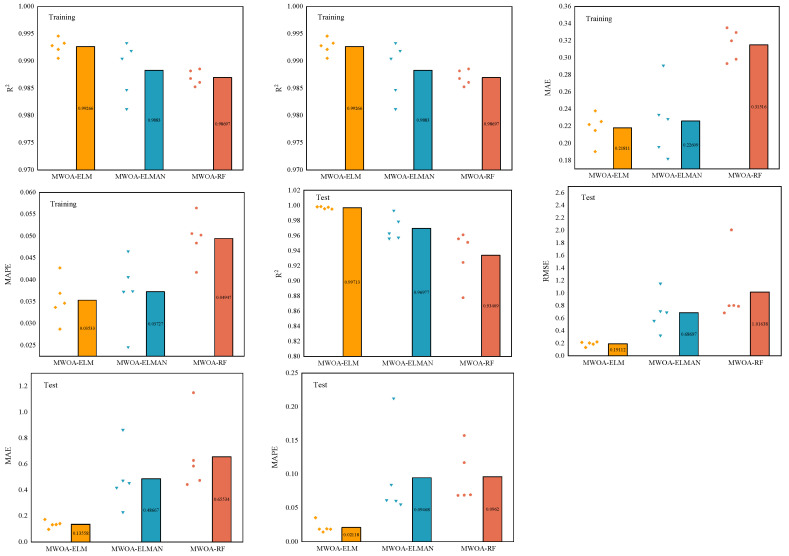
Box line plots of the Training and Testing sets for MWOA-ELM, MWOA-RF and MWOA-ELMAN models.

In Section 5, MWOA is proven to improve the generalization of the three models ELM, RF, and ELMAN. It is also possible to prove that the three optimization models are the most exact (it has been shown in the literature that a model is highly accurate when its *R*^2^ is more significant than 0.9 [88]). This indicates that ML techniques can meet the prediction accuracy. However, it is necessary to perform a comparative analysis to obtain the optimal model. Figure 11 represents the metric radar plots for the training and testing sets of the MWOA-ELM, MWOA-RF, and MWOA-ELMAN models. The figure shows that the MWOA-ELM model outperforms the other two models on the training and testing sets, with the highest *R*^2^ and lowest RMSE, MAE and MAPE. Figure 12 represents the Taylor diagrams for the training and testing sets of the three models. The MWOA-ELM model performs the best, with the lowest error metric on the Taylor diagram, while being closest to the best reference point. Table 8 shows the average of the 5-fold cross-validation results for each of the three models. MWOA-ELM model outperformed the training process during testing. In contrast, the prediction accuracy of both the MWOA-RF and MWOA-ELMAN models decreased, with the MWOAA-RF model decreasing the most (by about 5.6%), indicating that MWOA-ELM is very stable. Figure 13 represents box plots of the training and testing sets for the three models. The bars indicate the mean value of each model. The training and prediction results of the models can be seen more visually in the box plots, where the MWOA-ELM model is not necessarily the best on the training set but has the lowest mean value. In the testing set, it is the best performer and relatively stable, with all *R*^2^ around 0.99. This suggests that the MWOA-ELM is the best. It can also demonstrate that with the introduction of cross-validation, the model is very realistic in its calculations and avoids chance.

### 6.2. Sensitivity Analysis

Sensitivity factor analysis (SA) is an effective method for measuring the influence of model input parameters on output parameters. Sensitivity factor analysis provides feedback on the importance of the model input parameters. Therefore, this study uses the cosine amplitude method (CAM) [89] to perform sensitivity factor analysis on three models and an experimental model. The expressions are as follows:

(37) $$R_{ij}=\frac{\sum_{k=1}^{n}x_{ik}x_{jk}}{\sqrt{\sum_{k=1}^{n}x_{ik}^{2}\sum_{k=1}^{n}x_{jk}^{2}}}\;$$

where *x_i_* denotes input parameters; *x_j_* denotes output parameters; *n* denotes the number of data; *R_ij_* denotes the strength of the relationship.

**Figure 14:**
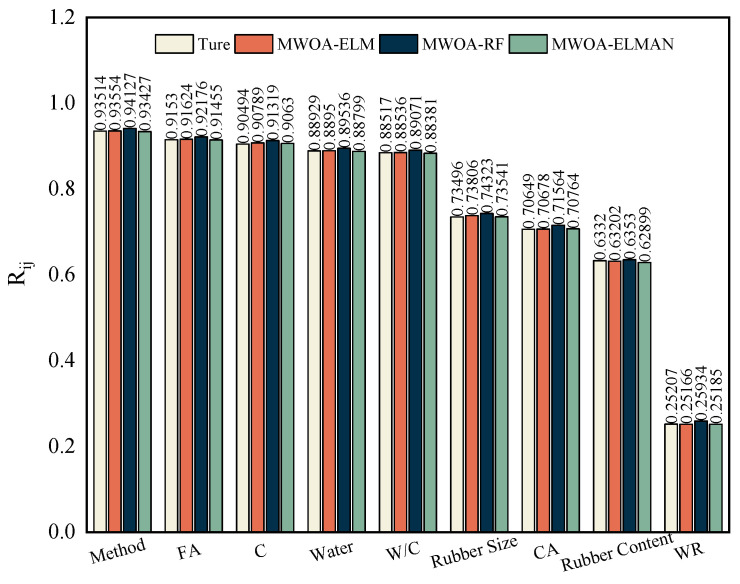
Sensitivity analysis of three models and experimental models.

Figure 14 represents the strength factor of the relationship between each variable and *D_CI_*. It can be seen that the three models show similar sensitivity to the experimental model, justifying the developed model. As seen from the graph, the measurement method has the most significant effect on the *D_CI_* of RC, followed by FA, while the impact of WR is the least.

### 6.3. Prediction of Typical Machine Learning Model

**Figure 15:**
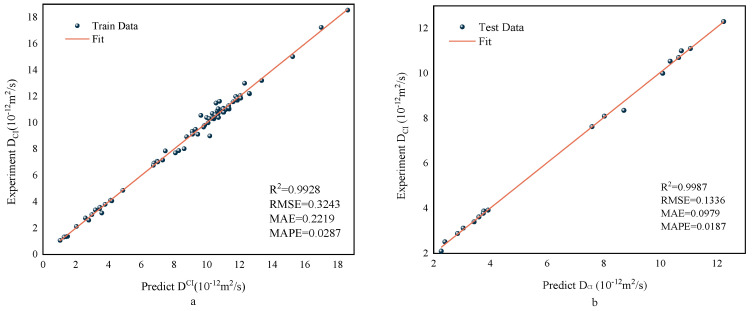
Regression results for the Training (**a**) and Testing (**b**) sets of the MWOA-ELM model.

**Figure 16:**
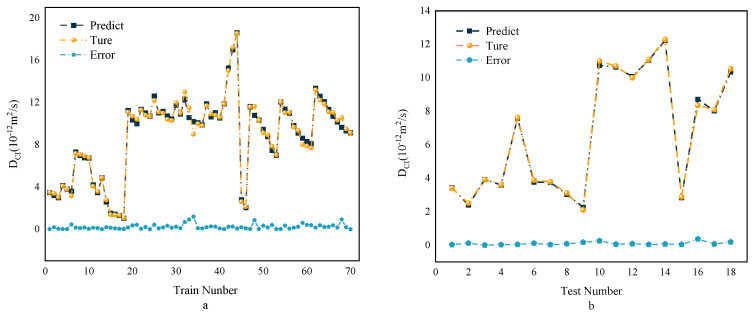
Comparison and error results between the Training (**a**) and Testing (**b**) sets of the MWOA-ELM model.

In Section 6.1, the MWOA-ELM model was proven to be the best model. Therefore, the typical predictions from the MWOA-ELM model are shown in this section. Figure 15 depicts the regression results for the training and testing set. It is important to emphasize that the MWOA-ELM model has strong predictive power. Its indicator values for the training and testing set were $R^{2}=0.9928$, RMSE = 0.3243, MAE = 0.2219, MAPE = 0.0287 and $R^{2}=0.9987$, RMSE = 0.1336, MAE = 0.0979, MAPE = 0.0187. Figure 16 shows the predicted values of the MWOA-ELM model compared to the actual values, with the error values included. The comparison results show that the predicted values of the *D_CI_* of RC are consistent with the experimental values. It is worth noting that the error between the training set and the testing set is small, which indicates that the MWOA-ELM model can predict the *D_CI_* of RC well. The above results suggest that predicting the *D_CI_* of RC using the MWOA-ELM model is feasible. This may contribute to developing a numerical tool for determining durability indicators for RC. The application of intelligent algorithms is equally effective. In the future, consider increasing the amount of data and input variables, which would improve the ability of the MWOA-ELM model to predict the *D_CI_* of RC. The weight matrix for the MWOA-ELM model’s typical prediction result is shown in Appendix A.

### 6.4. Forecast Comparison

**Figure 17:**
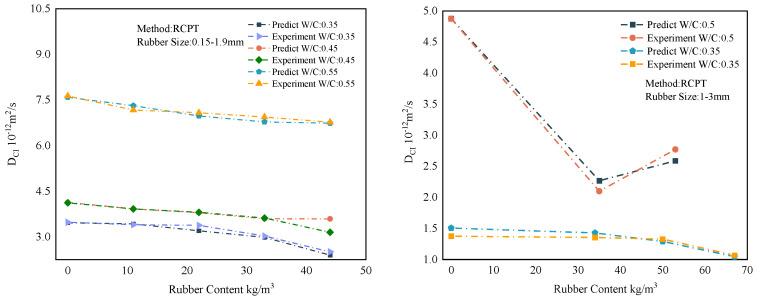
Prediction results of the MWOA-ELM model for the literature [54] (**Left**) and [55] (**Right**).

**Figure 18:**
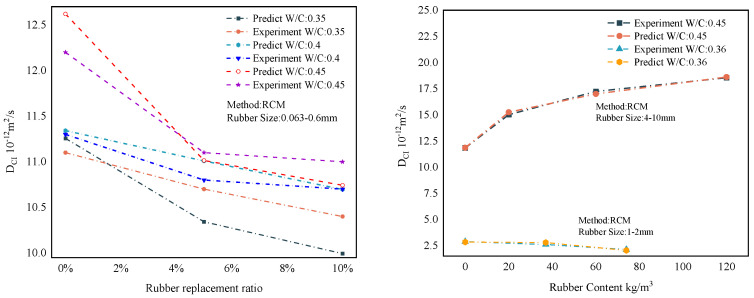
Prediction results of the MWOA-ELM model for the literature [57] (**Left**), [59] (**Right**, **Up**), and [58] (**Right**, **down**).

**Figure 19:**
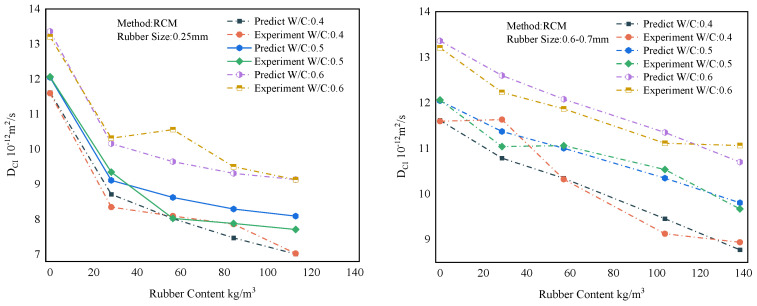
Prediction results of the MWOA-ELM model for the literature [56].

**Figure 20:**
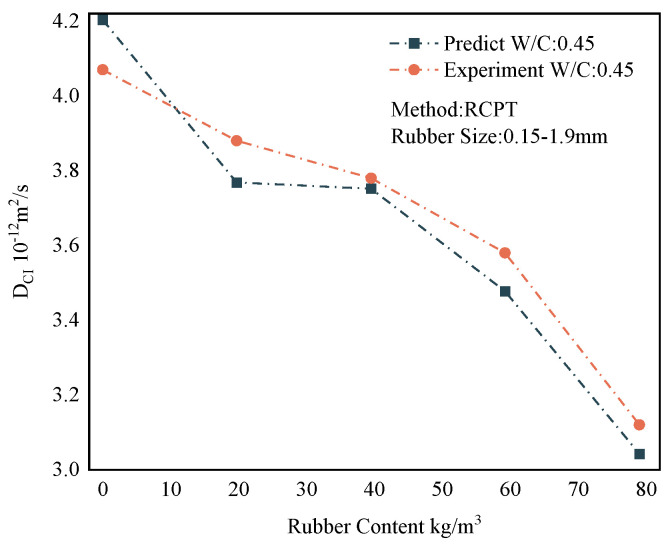
Prediction results of the MWOA-ELM model for the literature [22].

The overall results of the representative predictions of the MWOA-ELM model are shown in Section 6.3. However, it is necessary to show the forecasting results within the model. This provides a more intuitive view of the model’s predictions. Therefore, this section shows the prediction results of the MWOA-ELM model under different methods separately. Figure 17 represents the prediction result of the MWOA-ELM model for the literature [54,55]. The model’s predictions can be judged from Figure 17, in general agreement with the experimental results. Figure 18 represents the prediction results of the model for the literature [57,58,59]. It can be seen from Figure 18 that the model predicts better results for the literature [58,59]. The predicted curves for the literature [57], showed some deviations, but the overall trend was consistent. However, the errors are acceptable in terms of the overall results of Section 6.3. Figure 19 represents the prediction results of the model for the literature [56]. From Figure 19, it can be observed that the prediction curves both show some deviations. This may be due to the algorithm falling into the local optimum when optimizing this part of the data, making the model learn insufficiently. However, in general, the errors are still acceptable. Figure 20 reflects the predictions of the literature [22]. From Figure 20, the prediction trends are consistent and the errors are relatively small. The above conclusions indicate that using the MWOA-ELM model predicting the $D_{CI}$ of RC is feasible. The model can still be further optimized, which includes developing more powerful algorithms and increasing the amount of data.

### 6.5. Comparative Analysis with Other Models

**Figure 21:**
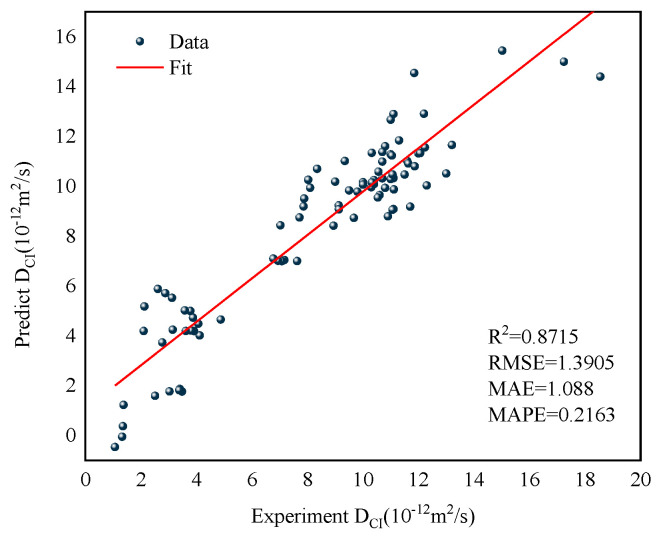
Regression analysis result of the MRL model.

To further verify the MWOA-ELM model’s reliability, this study introduces a multiple linear regression model for comparison [90]. Since the MRL model is similar to the ML model in that it also studies the effects of multi-factor interactions, it is compared with the MWOA-ELM model. Figure 21 represents the regression analysis results of the MRL model. The results of the evaluation indicators for the MWOA-ELM (Mean of overall model results under five-fold cross-validation) model and the MRL model are shown in Table 9. Obviously, MWOA-ELM is superior to the MRL model.

**Figure 22:**
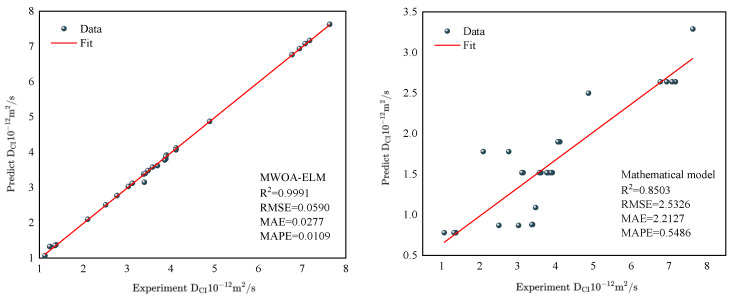
Regression analysis result of the MWOA-ELM and Mathematical model.

Comparison with other models is equally necessary. Ye [91] developed a mathematical model to predict the *D_CI_* of RC, because the input variables for the mathematical model used are the water-cement ratio, the rubber admixture, and the rubber size. Therefore, the input variables of the MWOA-ELM model are replaced in the same way. Keeping the same input variables is better for comparison. The data were obtained from three randomly selected papers to avoid complex calculation [22,54,55]. Figure 22 represents the results of the regression analysis for the two models. The results of the evaluation indicators for the two models are shown in Table 10. From Figure 22, the regression analysis result of the MWOA-ELM model is better, with the *R*^2^ of 0.991 higher than the mathematical model of 0.8053. Similar results are seen in the other error evaluation indicators in Table 9. This indicates that the MWOA-ELM model has better prediction and generalization ability.

## 7. Conclusions and Future Prospect

This study used ML techniques to predict the *D_CI_* of RC. Three models, ELM, RF, and ELMAN were developed to investigate. The established MWOA was also used to optimize the three models. Four metrics, RMSE, MAE, and MAPE, were used to evaluate the performance of the models. According to the prediction results, the MWOA-optimized ELM, RF, and ELMAN models successfully predicted the *D_CI_* of RC. At the same time, they had the highest *R*^2^ and lowest errors compared to the unoptimized models. This indicates that the algorithm established is valid. Comparing the three optimal models, the MWOA-ELM model performs the best. The three models were shown to have similar sensitivity to the experimental model by the CAM method. This justifies the developed models. Comparing the typical prediction results of the MWOA-ELM model with the actual values shows that the prediction results generally agree with the experiment, while the error is within a reasonable range. Comparison with the MRL and published mathematical model show that the MWOA-ELM model performs the best. This suggests that the MWOA-ELM model can accurately predict the *D_CI_* of RC.

In summary, this study successfully used ML techniques to predict the *D_CI_* of RC while demonstrating the proposed MWOA is valid. This provides a new option for determining the *D_CI_* of RC. However, observation of the results revealed that the proposed method could be further optimized to better understand RC’s chloride permeation process. This includes developing more robust algorithms, increasing the data set, adding input variables, and enhancing the interpretable analysis of the model.

## Figures and Tables

**Table 1:** Statistical analysis of input and output variables.

	Max	Min	Average	Median	Stdd	Stde
Method	2.00	1.00	1.6932	2.00	0.4612	0.4638
C·kg/m^3^	457.00	100.00	350.69	387.50	123.16	123.87
WR·kg/m^3^	5.82	0.00	0.62	0.00	1.34	1.35
W·kgm^3^/	272.00	35.00	157.32	161.90	65.19	65.57
W/C	0.60	0.35	0.44	0.45	0.076	0.078
FA·kg/m^3^	1360.00	174.00	862.76	1005.00	365.72	367.82
CA·kg/m^3^	1124.00	0.00	504.95	607.00	410.24	412.59
Rubber Size	5.00	0.00	1.4773	1.00	1.215	1.222
Rubber Content kg/m^3^	138.40	0.00	35.89	22.00	37.55	37.77
*D_CI_*·10^−12^m^2^	18.55	1.07	8.39	9.74	3.88	3.90

**Table 2:** Parameter settings for the MWOA-ELM model.

Parameter	Setting
Popsize	30
Maxgen	100
d1, d2	1 × 10^−4^
b	1
Hiddennum layer	28
Activation function	Sigmoid

**Table 3:** Average of evaluation indicators for three ELM models.

		*R* ^2^	RMSE	MAE	MAPE (%)
ELM	Train	0.9602	0.7691	0.6233	0.1132
	Test	0.6458	2.6232	1.4539	0.3509
WOA-ELM	Train	0.9848	0.4518	0.3475	0.0619
	Test	0.9390	0.8810	0.6584	0.1155
MWOA-ELM	Train	0.9927	0.3287	0.2181	0.0353
	Test	0.9971	0.1911	0.1356	0.0212

**Table 4:** Parameter settings for the MWOA-RF model.

Parameter	Setting
Popsize	30
Maxgen	100
Forest size	24
Number of leaves	8
Number of cross-validation	5
d1, d2	1 × 10^−4^
b	1

**Table 5:** Average of evaluation indicators for three RF models.

		*R* ^2^	RMSE	MAE	MAPE (%)
RF	Train	0.653	2.2463	1.2972	0.2766
	Test	0.5768	2.5709	1.7408	0.4015
WOA-RF	Train	0.9661	0.7709	0.5698	0.1009
	Test	0.8776	1.4409	1.0027	0.1941
MWOA-RF	Train	0.9870	0.4520	0.3152	0.0495
	Test	0.9341	1.0164	0.6553	0.0962

**Table 6:** Parameter settings for the MWOA-ELMAN model.

Parameter	Setting
Popsize	30
Maxgen	100
Hiddennum_best	13
Number of cross-validation	5
Activation function	tansig, purelin
Training function	trainlm
d1, d2	1 × 10^−4^
b	1

**Table 7:** Average of evaluation indicators for three ELMAN models.

		*R* ^2^	RMSE	MAE	MAPE (%)
ELMAN	Train	0.8275	1.6528	1.0820	0.1938
	Test	0.7108	2.1198	1.3609	0.2590
MWOA-ELMAN	Train	0.9783	0.5704	0.3207	0.0523
	Test	0.9390	0.8810	0.6584	0.1155
MWOA-ELMAN	Train	0.9883	0.4140	0.2261	0.0373

**Table 8:** Average of evaluation indicators for MWOA-ELM, MWOA-RF, and MWOA-ELMAN.

		*R* ^2^	RMSE	MAE	MAPE (%)
MWOA-ELM	Train	0.9927	0.3287	0.2281	0.0353
	Test	0.9971	0.1911	0.1356	0.0212
MWOA-RF	Train	0.9870	0.4520	0.3152	0.0495
	Test	0.9341	1.0163	0.6553	0.0962
MWOA-ELMAN	Train	0.9883	0.4141	0.2261	0.0373
	Test	0.9698	0.6870	0.4867	0.0947

**Table 9:** Evaluation indicators for the MWO-ELM and MRL model.

	*R* ^2^	RMSE	MAE	MAPE (%)
MWOA-ELM	0.9937	0.3064	0.2096	0.0325
MLR	0.8715	1.3905	1.088	0.2163

**Table 10:** Evaluation indicators for the MWO-ELM and Mathematical model.

	*R* ^2^	RMSE	MAE	MAPE (%)
MWOA-ELM	0.9991	0.0590	0.0277	0.0109
Ye [91]	0.8503	2.5326	2.2127	0.5486

## Data Availability

The data used in the article can be obtained from the author here.

## Appendix A

MWOA-ELM Model:

***IW1***=
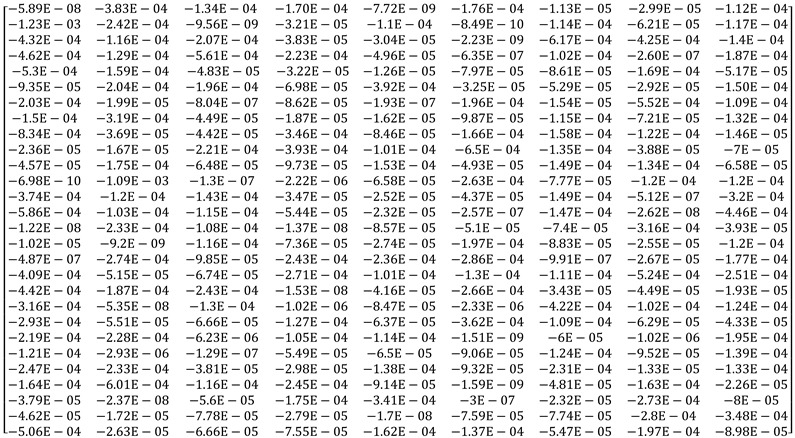
***B1***= [−3.11E−05 −7.13E−06 −1.01E−04 −8.49E−05 −2.3E−04 −9.25E−05 −1.48E−04 −1.28E−04 −1.44E−04 −1.19E−04 −2.24E−04 −1.95E−04 −3.91E−11 −1.62E−04 −2.03E−04 −2.39E−04 −6.9E−06 −7.85E−05 −2.04E−04 −2.79E−07 −5.29E−09 −1.3E−04 −1.04E−04 −1.97E−04 −3.65E−05 −2.34E−05 −9.78E−05 −1.09E−04 ]^T^
***LW1***= [−1.67E+11 −7.39E+11 −3.52E+11 0.37E+11 1.95E+11 −5.82E+11 1.71E+11 −2.19E+11 −1.96E+11 6.51E+11 −4.07E+11 −1.30E+11 1.13E+11 −2.14E+11 1.68E+11 1.61E+11 −2.45E+11 3.63E+11 −6.37E+11 −3.95E+11 −4.37E+11 8.69E+11 −1.1E+11 3.79E+11 1.42E+11 8.13E+11 −6.08E+11 −9.04E+11 ]^T^
